# APOBEC3G/3A Expression in Human Immunodeficiency Virus Type 1-Infected Individuals Following Initiation of Antiretroviral Therapy Containing Cenicriviroc or Efavirenz

**DOI:** 10.3389/fimmu.2018.01839

**Published:** 2018-08-08

**Authors:** Daniela A. Covino, Cristina Purificato, Laura Catapano, Clementina M. Galluzzo, Maria Cristina Gauzzi, Stefano Vella, Eric Lefebvre, Star Seyedkazemi, Mauro Andreotti, Laura Fantuzzi

**Affiliations:** ^1^National Center for Global Health, Istituto Superiore di Sanità, Rome, Italy; ^2^Allergan plc, South San Francisco, CA, United States

**Keywords:** APOBEC3A, APOBEC3G, antiretroviral therapy, cenicriviroc, chronic inflammation, disease progression

## Abstract

Apolipoprotein B mRNA editing enzyme catalytic polypeptide-like 3 (APOBEC3) family members are cytidine deaminases that play crucial roles in innate responses to retrovirus infection. The mechanisms by which some of these enzymes restrict human immunodeficiency virus type 1 (HIV-1) replication have been extensively investigated *in vitro*. However, little is known regarding how APOBEC3 proteins affect the pathogenesis of HIV-1 infection *in vivo* and how antiretroviral therapy influences their expression. In this work, a longitudinal analysis was performed to evaluate APOBEC3G/3A expression in peripheral blood mononuclear cells of antiretroviral-naive HIV-1-infected individuals treated with cenicriviroc (CVC) or efavirenz (EFV) at baseline and 4, 12, 24, and 48 weeks post-treatment follow-up. While APOBEC3G expression was unaffected by therapy, APOBEC3A levels increased in CVC but not EFV arm at week 48 of treatment. APOBEC3G expression correlated directly with CD4^+^ cell count and CD4^+^/CD8^+^ cell ratio, whereas APOBEC3A levels inversely correlated with plasma soluble CD14. These findings suggest that higher APOBEC3G/3A levels may be associated with protective effects against HIV-1 disease progression and chronic inflammation and warrant further studies.

## Introduction

Highly active antiretroviral therapy dramatically reduced human immunodeficiency virus type 1 (HIV-1)-related morbidity and mortality and currently suppresses viral replication in the majority of compliant patients. Antiretroviral drug regimens usually containing three active drugs from two or more classes are recommended for virologic suppression (1). Initial drug combinations generally consist of two nucleoside reverse transcriptase inhibitors plus a third drug such as an integrase inhibitor, a non-nucleoside reverse transcriptase inhibitor, or a protease inhibitor. In case of virologic failure or multi-class resistance, drugs not generally recommended for initial therapy can be considered, such as CC chemokine receptor 5 (CCR5) antagonists. Despite viral suppression, signs of inflammation and immune activation persist in most patients (2–6). Cenicriviroc (CVC) is a small-molecule CCR5 antagonist which has completed phase II of clinical development in HIV-1 infection (7, 8). It also inhibits CCR2, a receptor for CC chemokine ligand 2 (CCL2). The CCR2/CCL2 axis has been associated with various inflammatory diseases as well as with the high level of immune activation/inflammation and virus-associated disorders in HIV-1-infected individuals (9–12).

The apolipoprotein B mRNA editing enzyme catalytic polypeptide-like 3 (APOBEC3; A3) proteins are cytidine deaminases playing a crucial role in antiviral innate immunity (13, 14). Among the seven human A3 enzymes (from A to H), A3G was identified as the cellular restriction factor responsible for inhibiting HIV-1 replication in the absence of the virally encoded protein virion infectivity factor (15). It is encapsidated into virions and blocks HIV-1 replication upon entry in newly infected cells mainly by causing C-to-U deamination on the single stranded viral DNA during reverse transcription, leading to either the hypermutation of the viral genome or the degradation of viral DNA by cellular repair mechanisms. Other family members, such as A3F and A3H, can be encapsidated into budding virions and exert their antiviral activity in newly infected cells. Conversely, A3A is the only A3 enzyme which can restrict infection directly in the target cells where it is endogenously expressed (16). The A3 proteins are expressed in a tissue- and cell type-specific manner (17–19). Peripheral blood leukocytes express transcripts for all the family members, with A3G and A3A being the most highly represented (18). The former is greatly expressed in CD4^+^ T lymphocytes and myeloid cells (20), whereas expression of the latter is quite specific to myeloid lineage cells (21–24). While much has been learned about A3 proteins and their roles in HIV-1 restriction *in vitro*, little is known about how they impact on the *in vivo* pathogenesis of HIV-1 in the host (25, 26). Although some studies investigated A3 expression (mainly A3G and A3F) and activity in HIV-1^+^ subjects and the correlations with clinical parameters of infection, scattered data are available on the impact of antiretroviral therapy. The aim of this study was to assess the expression of A3G and A3A in patients treated with CVC or conventional therapy and evaluate its association with virological, immunological, and inflammatory parameters.

## Materials and Methods

### Study Patients and Ethical Issues

This study was exempt from ethics approval since it involved the secondary use of stored anonymized biological material from a subset of HIV-1^+^ patients enrolled in Study 202 (ClinicalTrials.gov NCT01338883), a multicenter 48-week phase 2b trial comparing treatment with CVC at two different doses (100 and 200 mg) versus efavirenz (EFV) 600 mg, both in combination with emtricitabine/tenofovir disoproxil fumarate, in antiretroviral treatment-naive, HIV-1-infected adults with CCR5-tropic virus (7). Study 202 was conducted in accordance with the Declaration of Helsinki, was approved by central or local institutional review boards or ethics committees at each study site, and a written informed consent was obtained from study participants. The trial included the measurement of biomarkers associated with inflammation and immune activation and sample storage for possible future studies. Available cryo-preserved peripheral blood mononuclear cell (PBMC) samples collected at baseline and after 4, 12, 24, and 48 weeks of treatment from participants who completed the follow-up period were used.

### Western Blot Analysis of A3 Proteins

A3G and A3A expression was determined by western blot. Whole cell extracts were obtained by lysing PBMCs in RIPA buffer [150 mM NaCl, 50 mM Tris–Cl (pH 7.5), 1% Nonidet P-40, 0.5% sodium deoxycholate, and 0.1% sodium-dodecyl sulfate (SDS)] containing a cocktail of protease (Roche, Basel, Switzerland) and phosphatase inhibitors (Sigma-Aldrich, Milan, Italy) as previously described (21). Protein concentration was determined using the Bradford reagent (Bio-Rad, Milan, Italy) and a standard curve obtained with bovine serum albumin (Bio-Rad). Cell lysates (20 µg per lane) were fractionated on 10–12% SDS-PAGE and electroblotted to nitrocellulose filters (Protran BA 85, Schleicher & Schuell, Keene, Netherlands). A reference curve with dose-scale concentration (20–10–5 μg) of protein extracts derived from healthy donor PBMCs was used to assess the best primary and secondary antibody (Ab) dilutions and was included in each blot (data not shown). Membranes were incubated with 4–5% fat-free milk dissolved in PBS-T (PBS 1×, 0.05%) to block non-specific binding and then probed with the following Abs: anti-human A3A (rabbit polyclonal D23, Santa Cruz Biotechnology, Santa Cruz, CA, USA), anti-human A3G (rabbit polyclonal D9C6Z, Cell Signaling Technology, Beverly, MA, USA), and anti-actin (mouse monoclonal Abs-5, BD Biosciences, San Diego, CA, USA) as gel loading control. In some patients, A3G/A3A expression was confirmed by using a rabbit polyclonal anti–human A3G/A3A serum kindly provided by Dr. M. Malim (data not shown) (27). Blots were then incubated with appropriate secondary Abs conjugated with horseradish peroxidase (Santa Cruz Biotechnology) followed by Amersham ECL Western blot detection Reagent (GE Healthcare Life Sciences, Pittsburgh, PA, USA) or Pierce SuperSignal West Femto Substrate (Thermo Fisher Scientific, Waltham, MA, USA) according to the manufacturer’s instructions. Levels of A3A, A3G, and actin proteins were detected and quantified by using Chemidoc XRS (Bio-Rad).

### Measurement of Virological, Immunological, and Inflammatory Parameters

Human immunodeficiency virus type 1 RNA levels, blood CD4^+^ and CD8^+^ cell counts, immune activation (CD3/CD4/CD38 and CD3/CD8/CD38), and inflammatory [high sensitivity C-reactive protein (hs-CRP), D-dimer, fibrinogen, and soluble CD14 (sCD14)] biomarkers were assessed at baseline and after 4 (except for sCD14), 12, 24, and 48 weeks of treatment. Viral load was quantified by the TaqMan assay (Applied Biosciences, Life Technologies, Carlsbad, CA, USA). The immune activation marker CD38 was measured by flow cytometry using cryo-preserved PBMCs. D-dimer was quantified by immunoturbidimetric assay using a D-dimer assay (Liatest) kit (Diagnostica Stago, Asnieres, France), hs-CRP by immunochemiluminometric assay using a quantitative C-reactive protein kit (Roche Diagnostics), and fibrinogen by polymerization function by the Clauss method using the Stago Fibrinogen kit (Diagnostica Stago); these biomarkers were measured by LabCorp Clinical Trials (Cranford, NJ, USA). sCD14 was quantified by R&D Systems using a solid phase sandwich ELISA with a human sCD14 Quantikine ELISA kit (R&D Systems, Minneapolis, MN, USA). All the parameters used in the correlation analysis were previously reported (7).

### Statistical Analysis

Intra-group variations between time points were assessed using Wilcoxon signed-rank tests, while the comparison between groups was done by the Mann–Whitney *U* test. Non-parametric Spearman tests were used to determine correlation coefficients. Observations were considered statistically significant when *p* < 0.05. SPSS version 24 (IBM Corp., Armonk, NY, USA) and GraphPad Prism version 7 (GraphPad Software, Inc., San Diego, CA, USA) were used for statistical analyses and graphs drawing.

## Results

**Figure 1:**
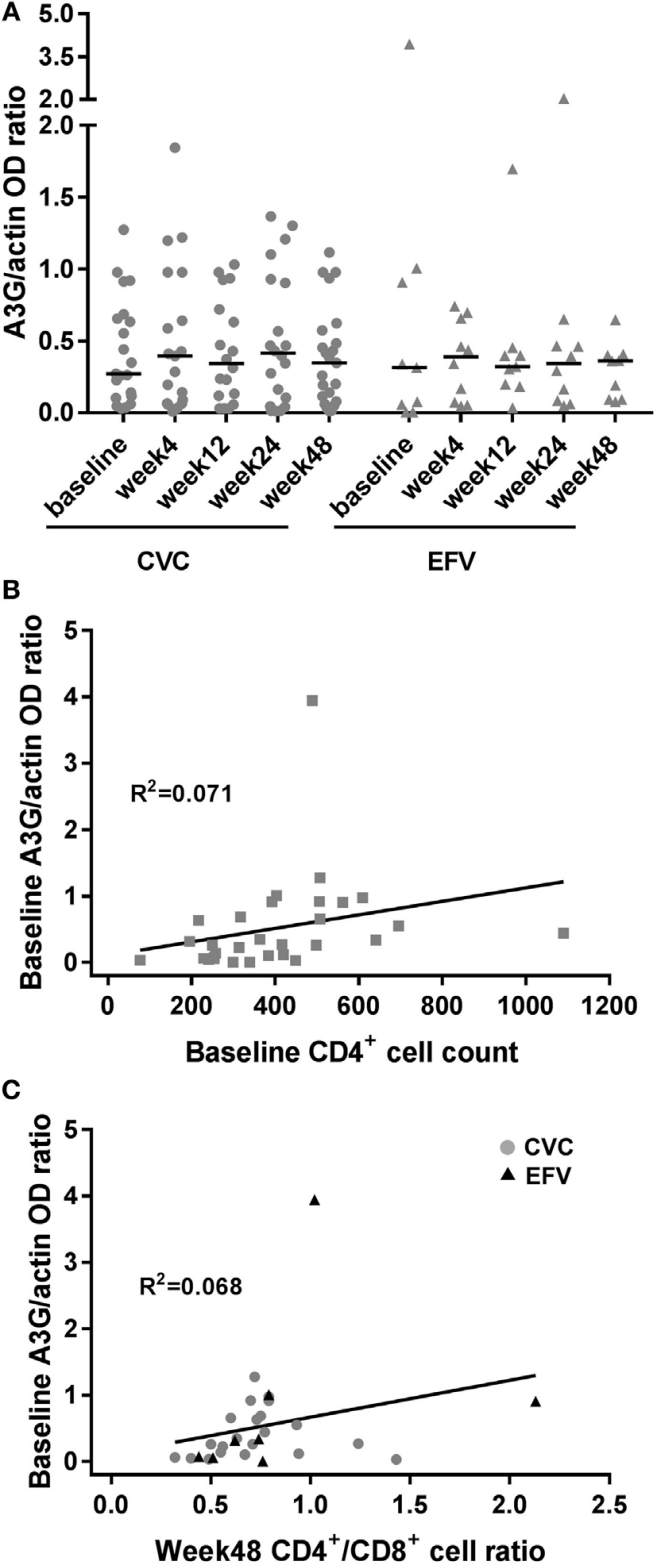
A3G expression in cenicriviroc (CVC) and efavirenz (EFV) arms and correlation with surrogate markers of disease progression. **(A)** Expression of A3G in peripheral blood mononuclear cells of subjects treated with CVC (circles) or EFV (triangles). The dot plots show the ratios of A3G to actin OD determined by densitometry for each time point of the patients analyzed (CVC: *n* = 21 baseline, *n* = 19 week 4, *n* = 18 week 12, *n* = 20 week 24, *n* = 23 week 48; EFV: *n* = 9 baseline, *n* = 10 week 4, *n* = 9 week 12, *n* = 10 week 24, *n* = 9 week 48). Median values (50th percentiles) are shown by the horizontal bars. **(B)** Correlation between baseline A3G levels and CD4^+^ cell counts (*n* = 30). **(C)** Correlation between baseline A3G levels and week 48 CD4^+^/CD8^+^ cell ratio (*n* = 29; CVC: *n* = 21; EFV: *n* = 8). Gray circles and black triangles, CVC- and EFV-treated subjects, respectively. Statistical analysis was done using the Wilcoxon signed-rank test **(A)** and the non-parametric one-tailed Spearman’s test to determine correlation coefficients **(B,C)**.

**Figure 2:**
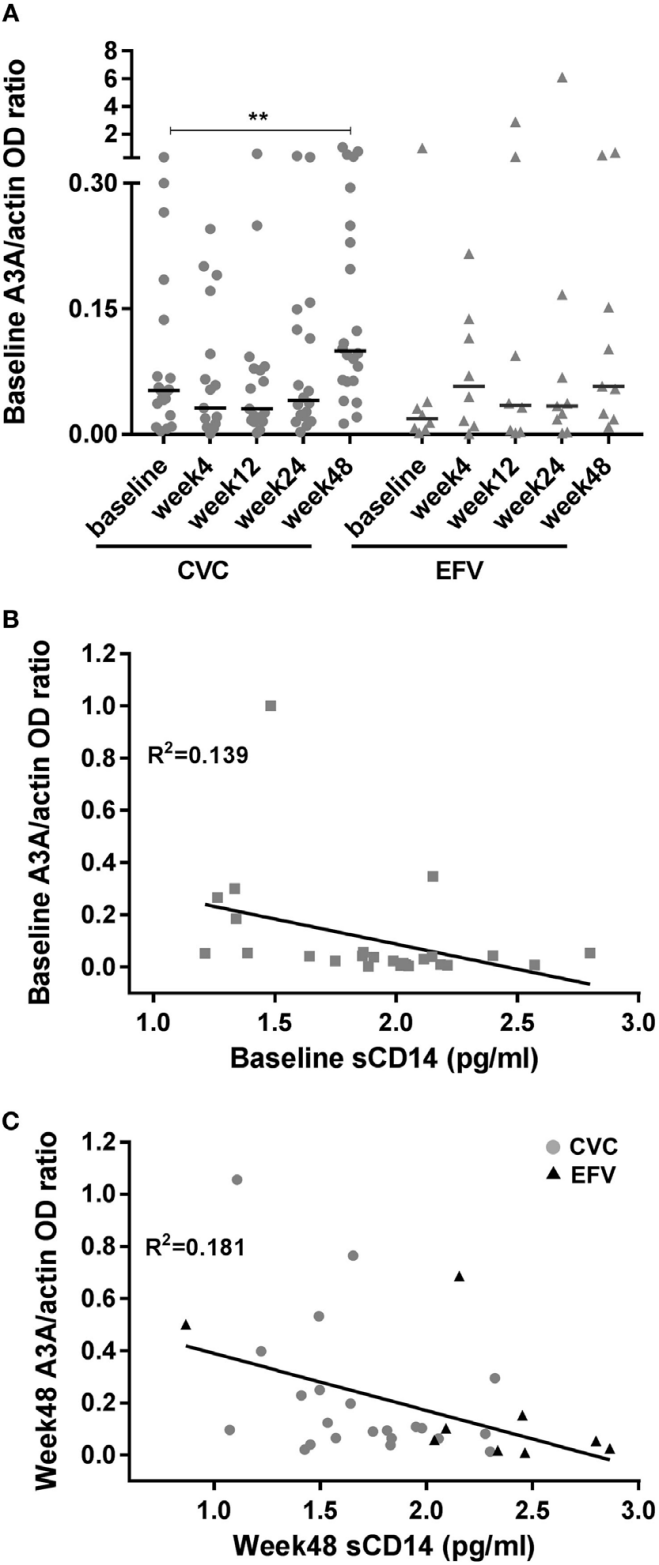
A3A expression in cenicriviroc (CVC) and efavirenz (EFV) arms and correlation with the biomarker of inflammation soluble CD14 (sCD14). **(A)** Expression of A3A in peripheral blood mononuclear cells of subjects treated with CVC (circles) or EFV (triangles). The dot plots show the ratios of A3A to actin OD determined by densitometry for each time point of the patients analyzed (CVC: *n* = 20 baseline, *n* = 17 week 4, *n* = 17 week 12, *n* = 18 week 24, *n* = 22 week 48; EFV: *n* = 8 baseline, *n* = 8 week 4, *n* = 8 week 12, *n* = 9 week 24, *n* = 9 week 48). Median values (50th percentiles) are shown by the horizontal bars. **p = 0.001 (week 48 versus baseline). **(B)** Correlation between baseline A3A and sCD14 levels (*n* = 25). **(C)** Correlation between week 48 A3A and sCD14 levels (*n* = 31; CVC: *n* = 22; EFV: *n* = 9). Gray circles and black triangles, CVC- and EFV-treated subjects, respectively. Statistical analysis was done using the Wilcoxon signed-rank test **(A)** and the non-parametric one-tailed Spearman’s test to determine correlation coefficients **(B,C)**.

We have performed a longitudinal analysis of A3G and A3A expression in PBMCs from 41 Study 202 participants (26 from the CVC 200 arm and 15 from the EFV arm). Demographic and baseline clinical characteristics prior therapy initiation of the HIV-1-infected subjects analyzed are provided in Table 1. Total cell extracts were obtained from patient PBMCs collected before and 4, 12, 24, and 48 weeks after therapy initiation, and A3G and A3A protein levels were assessed by western blot and normalized to actin by densitometric analysis. A3G was detected in all the subjects analyzed, whereas A3A protein was under the detection limit of the assay in 5 out of 41 patients (2 and 3 in CVC and EFV arms, respectively). Expression of both A3 proteins at baseline did not significantly differ between the two arms (Table S1 in Supplementary Material). As shown in Figure 1A, expression of A3G was not altered over the 48 weeks in both treatment arms. Conversely, A3A levels were significantly increased at week 48 of treatment in the CVC arm (*p* = 0.001), whereas no significant differences were observed in the EFV arm (Figure 2A).

**Table 1:** Demographic and baseline clinical characteristics of study subjects.

Variable	CVC 200 mg (*n* = 26)	EFV 600 mg (*n* = 15)	All (*n* = 41)	*p*^a^
**Male sex**
Number (percentage)	26 (100)	12 (80)	38 (93)	0.023
**Age**				
Years, median (range)	38 (21–57)	35 (20–49)	37 (20–57)	0.255
**Race** Number (percentage)				
Black/African-American	2 (8)	4 (27)	6 (15)	0.414
White	20 (77)	11 (73)	31 (76)	0.106
Other	4 (15)	0 (0)	4 (10)	n.a.
**Ethnicity** Number (percentage)				
Hispanic	12 (46)	5 (33)	17 (41)	0.090
Not Hispanic	14 (54)	10 (67)	24 (56)	0.414
**Viral load**				
log_10_ RNA copies/mL Median (range)	4.65 (3.55–5.37)	4.28 (3.35–5.49)	4.55 (3.35–5.49)	0.357
**CD4^+^ count**				
Cells/μL, median (range)	385 (77–1,090)	313 (191–641)	364 (77–1,090)	0.570
**CD8^+^ count**				
Cells/μL, median (range)	970 (407–2,915)	843 (394–1,734)	912 (394–2,915)	0.343
**CD4^+^/CD8^+^ ratio**				
Median (range)	0.40 (0.10–1.10)	0.40 (0.20–0.90)	0.40 (0.10–1.10)	0.773
**sCD14**				
pg/mL, median (range)	1.75^b^ (1.21–2.79)	2.03 (1.21–2.61)	1.93 (1.21–2.79)	0.062

*CVC, cenicriviroc; EFV, efavirenz; n.a., not applicable; sCD14, soluble CD14*.

*^a^Calculated by Mann–Whitney *U* test (continuous variables) or exact Pearson χ^2^ test (categorical variables)*.

*^b^Baseline sCD14 was available for 23 subjects in the CVC 200 mg arm*.

**Figure 3:**
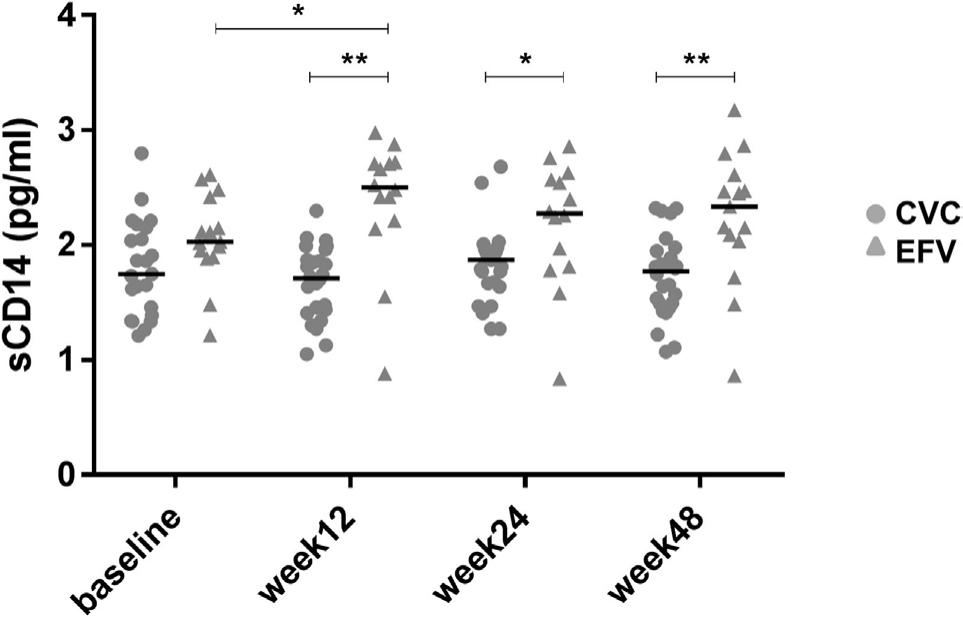
Levels of soluble CD14 (sCD14) in cenicriviroc (CVC) and efavirenz (EFV) arms. The dot plots show plasma sCD14 levels measured by ELISA at each time point of the patients analyzed (CVC: *n* = 23 baseline, *n* = 26 week 12, *n* = 25 week 24, *n* = 26 week 48; EFV: *n* = 15 baseline, *n* = 14 week 12, *n* = 14 week 24, *n* = 15 week 48). Median values (50th percentiles) are shown by the horizontal bars. Statistical analysis was done using the Mann–Whitney *U* or Wilcoxon signed-rank tests for unpaired or paired data. *p < 0.05; **p < 0.005.

To ascertain whether the observed levels of A3G/A3A expression had any clinical significance, the subjects’ viral loads, CD4^+^ and CD8^+^ cell counts and CD4^+^/CD8^+^ cell ratios, measured at baseline and at the end of the follow-up period (week 48), were examined as surrogate markers of disease progression. Table 2 summarizes these clinical parameters of study subjects during the treatment follow-up period. Spearman’s correlation was used to estimate the association between these biomarkers and A3G or A3A protein levels. No correlation was found between A3G or A3A expression and viral load (data not shown). Baseline A3G levels correlated directly with baseline CD4^+^ cell counts (coefficient = 0.541; *p* = 0.002) as well as CD4^+^/CD8^+^ cell ratios at week 48 of treatment (coefficient = 0.451; *p* = 0.01) (Figures 1B,C). Conversely, no correlation was found between A3A expression and these parameters or monocyte counts (data not shown). Interestingly, baseline A3A levels inversely correlated with baseline sCD14 (coefficient = −0.435; *p* = 0.03), and this effect was even stronger at week 48 of treatment (coefficient = −0.46; *p* = 0.009) (Figures 2B,C). As shown in Figure 3, levels of sCD14 were significantly higher in EFV-treated subjects compared to CVC arm at weeks 12, 24, and 48 of treatment but not at baseline (see Table 1). Moreover, sCD14 levels were significantly increased at week 12 of treatment in the EFV arm (*p* = 0.026), whereas no changes were observed during the follow-up period in the CVC arm. No correlation was observed between A3A expression and other non-specific markers of inflammation, namely hs-CRP, D-dimer, and fibrinogen, as well as the activation marker CD38 expressed on CD4^+^ and CD8^+^ lymphocytes (data not shown), which did not differ across the arms during follow-up (see Table S2 in Supplementary Material).

**Table 2:** Clinical parameters of study subjects during treatment follow-up.

Variable	Time point of follow-up	CVC 200 mg (*n* = 26)	EFV 600 mg (*n* = 15)	All (*n* = 41)	*p*^a^
**Viral load** log_10_ RNA copies/mL Median (range)	Week 4	2.51 (1.28–2.97)	2.03 (1.28–3.27)	2.30 (1.28–3.27)	0.066
	Week 12	1.60 (1.28–2.35)	1.28^b^ (1.28–2.03)	1.49 (1.28–2.35)	0.040
	Week 24	1.28 (1.28–1.90)	1.28 (1.28–2.01)	1.28 (1.28–2.01)	0.680
	Week 48	1.28 (1.28–1.96)	1.28 (1.28–1.60)	1.28 (1.28–1.96)	0.860

**CD4^+^ count** Cells/μL Median (range)	Week 4	439 (174–1,045)	441 (242–853)	439 (174–1,045)	0.935
	Week 12	526 (213–1,339)	501 (180–641)	506 (180–1,339)	0.159
	Week 24	543 (268–1,512)	405 (222–697)	481 (222–1,512)	0.261
	Week 48	573 (316–1,205)	501^c^ (332–907)	548 (316–1,205)	0.183

**CD8^+^ count** Cells/μL Median (range)	Week 4	1,005 (430–2,196)	737 (474–1,180)	878 (430–2,196)	0.028
	Week 12	934 (431–1,611)	814 (308–1,067)	823 (308–1,611)	0.042
	Week 24	932 (438–1,597)	701 (393–1,015)	765 (393–1,597)	0.012
	Week 48	831 (466–1,756)	797^c^ (380–1,323)	829 (380–1,756)	0.294

**CD4^+^/CD8^+^ ratio** Median (range)	Week 4	0.45 (0.10–1.20)	0.60 (0.40–0.90)	0.50 (0.10–1.20)	0.100
	Week 12	0.60 (0.20–1.40)	0.60 (0.40–1.60)	0.60 (0.20–1.60)	0.493
	Week 24	0.60 (0.20–1.40)	0.60 (0.50–1.70)	0.60 (0.20–1.70)	0.574
	Week 48	0.70 (0.30–1.40)	0.60^c^ (0.40–2.10)	0.70 (0.30–2.10)	0.616

*CVC, cenicriviroc; EFV, efavirenz*.

*^a^Calculated by Mann–Whitney U test*.

*^b^Week 12 HIV-1 RNA was available for 14 subjects in the EFV 600 mg arm*.

*^c^Week 48 CD4^+^ and CD8^+^counts were available for 14 subjects in the EFV 600 mg arm*.

## Discussion

The role of A3s in regulating HIV-1 replication *in vivo* is unclear. Previous studies have addressed A3G (and A3F) expression and function in PBMCs of HIV-1^+^ subjects by quantifying A3 transcripts or editing in HIV-1 DNA. A positive correlation of A3G mRNA levels and/or viral DNA hypermutation with CD4^+^ cell count of HIV-1^+^ subjects and a negative correlation with viral load were identified in some studies (28–32) but not in others (33, 34). Higher levels of A3G were also observed in HIV-1-exposed uninfected individuals compared to healthy control or infected subjects (28, 35) and in long-term non-progressors compared to HIV-1-uninfected individuals and normal progressors (30). However, not all the studies were concordant with these associations (36), and higher A3G levels were found in HIV-1^−^ compared to HIV-1^+^ individuals, including matched pre- and post-infection samples from the same subjects, suggesting that A3G transcription may be downregulated upon infection (33). These contrasting results might be due to either technical issues or the size/characteristics of the cohorts studied. In this study, we conducted a longitudinal *ex vivo* analysis of A3G and A3A protein expression in PBMCs of HIV-1-infected individuals at baseline and at various times of antiretroviral therapy with CVC or EFV. Our results highlight a direct correlation between baseline A3G levels and either baseline CD4^+^ cell counts, as previously reported by others (28, 29, 37), or CD4^+^/CD8^+^ cell ratios at week 48 of treatment. The former is a well-known predictor of disease progression, and even the latter has been proposed in recent years as a marker of immune dysfunction, viral reservoir size, and a prognostic indicator for non-AIDS mortality (38). Although correlation does not necessarily indicate causation, these results support the hypothesis that enhanced A3G expression, even if not correlating with viral load, may provide protective effects against disease progression through the association with either increased CD4^+^ cell count in therapy naive patients or CD4^+^/CD8^+^ cell ratio during antiretroviral treatment.

To our knowledge, this study is the first to show that therapy initiation with diverse antiretroviral regimens can differently affect the expression of A3 proteins. Indeed, treatment with CVC, but not with EFV, determined an increase of A3A, but not A3G, expression. Although type I IFNs are the best characterized inducers of A3A expression, its role in the modulation of A3A levels in CVC-treated subjects was not investigated in this study. However, other stimuli have been shown to modulate the expression of this protein in an IFN-independent manner (19). In particular, our group demonstrated that endogenous CCL2 represents an autocrine factor acting as a negative regulator of A3A expression in macrophages. Indeed, the neutralization of this chemokine determined a specific increase in A3A, but not A3G, expression in either uninfected or HIV-1-infected monocyte-derived macrophages, and this effect was associated with a decrease of viral replication. Treatment with anti-IFN-α/β serum demonstrated that CCL2 blocking-mediated A3A induction was type I IFN independent (21, 39). Thus, the increase of A3A levels induced by CVC treatment may be a consequence of the CCR2 antagonistic activity of the drug. This is further supported by the lack of induction of A3G expression following CVC treatment. These results add new insights into the notion that blocking the CCR2/CCL2 axis may regulate the expression of this innate intracellular viral antagonist. Unlike A3G, *in vivo* A3A expression in HIV-1^+^ subjects has been poorly investigated. A3A transcripts were shown to be downregulated after initiation of antiretroviral therapy in whole blood samples from 10 Ugandans with AIDS (40). In addition, A3A baseline expression in PBMCs from HIV-1-infected patients was found to significantly correlate with viral load decline observed following 3 weeks of treatment with pegylated IFN-α-2a (41). Although in the patients’ cohort analyzed in this study A3A levels do not correlate with either viral load or CD4^+^/CD8^+^ cell counts and ratios, an inverse correlation was found between A3A expression and sCD14 levels. CD14 is a coreceptor for LPS and its soluble form is a marker of monocyte activation, and represents an independent predictor of morbidity and mortality in people with HIV-1 infection (2). When considering the association between A3A and sCD14 within the two arms, we did not detect a significant correlation (data not shown), likely due to the limited number of subjects in each arm.

Chronic inflammation is considered nowadays a driving force of immune dysfunction and AIDS progression. A residual chronic immune activation persists even in HIV-1-infected patients in which viral replication is inhibited by antiretroviral therapy. In fact, persistent latently infected cells contribute to the continuous activation of immune cells, establishing a dangerous vicious cycle between viral persistence and immune activation which contributes to the development of pathological conditions and hinders a complete remission (2). Although virologic success and CD4^+^ cell count increase were similar across CVC and EFV arms (7), lower levels of sCD14 were present in the CVC-treated group, suggesting the potential anti-inflammatory effects of this drug. This was confirmed in a very recent study in which virally suppressed chronic HIV-1-infected individuals were treated with CVC for 24 weeks (42). Despite the power of our study was limited by the small number of subjects analyzed, the increased expression of A3A in the CVC-treated group suggests an unprecedented link between decreased inflammation and innate antiviral responses. A3A is mainly expressed by monocytes, but the low amount of patient’s cells available imposed to perform the analysis of A3A levels on whole PBMCs. Additional work with larger cohorts and purified leukocyte populations is needed to robustly define the association between A3A expression and inflammation.

## Ethics Statement

This study was exempt from ethics approval since it involved the secondary use of stored anonymized biological material from a subset of HIV-1^+^ patients enrolled in Study 202 (ClinicalTrials.gov NCT01338883), a multicenter 48-week phase 2b trial comparing treatment with CVC at two different doses (100 and 200 mg) versus efavirenz (EFV) 600 mg, both in combination with emtricitabine/tenofovir disoproxil fumarate, in antiretroviral treatment-naive, HIV-1-infected adults with CCR5-tropic virus (7). Study 202 was conducted in accordance with the Declaration of Helsinki, was approved by central or local institutional review boards or ethics committees at each study site, and a written informed consent was obtained from study participants. The trial included the measurement of biomarkers associated with inflammation and immune activation and sample storage for possible future studies. Available cryo-preserved peripheral blood mononuclear cell (PBMC) samples collected at baseline and after 4, 12, 24, and 48 weeks of treatment from participants who completed the follow-up period were used.

## Author Contributions

DC designed and performed experiments, analyzed and interpreted the data, designed the figures, and contributed to write the manuscript. CP, LC, and CG performed experiments. MG contributed to data interpretation and manuscript writing. SV provided scientific advice and edited the manuscript. EL and SS contributed patients samples and clinical data. MA and LF conceived and designed the study, analyzed and interpreted the data, and wrote the manuscript. All the authors reviewed and approved the final manuscript.

## Conflict of Interest Statement

EL and SS are employees of Allergan. All other authors: no potential conflicts of interest. The reviewer LL and handling Editor declared their shared affiliation.
