# Supplementary material for: APOBEC3G/3A Expression in Human Immunodeficiency Virus Type 1-Infected Individuals Following Initiation of Antiretroviral Therapy Containing Cenicriviroc or Efavirenz
Source: Front Immunol. 2018 Aug 8;9:1839. doi: 10.3389/fimmu.2018.01839 (PMC6092507; doi:10.3389/fimmu.2018.01839)
Supplement: Supplementary file 1 [file Table_1.docx]

**Supplementary Table 1. Comparison of baseline A3G and A3A expression between CVC and EFV arms.**

| **Variable** | **CVC 200mg** | **EFV 600mg** | **All** | **p value^a^** |
| --- | --- | --- | --- | --- |
|  |  |  |  |  |
| Median A3G expression^b^  ratio A3G/actin OD  (minimum–maximum) | 0.27  (0.03-1.27) | 0.32  (0.006-3.95) | 0.29  (0.006-3.95) | 0.73 |
| Median A3A expression^c^  ratio A3A/actin OD  (minimum–maximum) | 0.05  (0.004-0.35) | 0.02  (0.002-0.99) | 0.04  (0.002-0.99) | 0.06 |

Abbreviations: CVC, cenicriviroc; EFV, efavirenz.

**^a^**Calculated by Mann–Whitney U test.

^b^Baseline A3G was available for 21 subjects in the CVC 200mg arm and 9 subjects in the EFV 600mg arm.

^c^Baseline A3A was available for 20 subjects in the CVC 200mg arm and 8 subjects in the EFV 600mg arm.
