# Supplementary material for: APOBEC3G/3A Expression in Human Immunodeficiency Virus Type 1-Infected Individuals Following Initiation of Antiretroviral Therapy Containing Cenicriviroc or Efavirenz
Source: Front Immunol. 2018 Aug 8;9:1839. doi: 10.3389/fimmu.2018.01839 (PMC6092507; doi:10.3389/fimmu.2018.01839)
Supplement: Supplementary file 2 [file Table_2.docx]

**Supplementary Table 2. Activation/inflammation markers of study subjects during follow-up.**

|  |  | |  | |  | |  |  |
| --- | --- | --- | --- | --- | --- | --- | --- | --- |
| **Variable** | | **Time point**  **of follow-up** | **CVC 200mg**  **(n=26)** | **EFV 600mg**  **(n=15)** | | **All**  **(n=41)** | | **p^a^** |
| **CD4^+^CD38^+^ cells^b^**  percentage  median (range) | | **Baseline** | 13.13  (2.92-44.10) | 12.20  (2.67-33.28) | | 12.42  (2.67-44.10) | | 0.245 |
|  |  | **Week 4** | 14.20  (2.63-39.90) | 11.18  (2.71-23.15) | | 12.90  (2.63-39.90) | | 0.372 |
|  |  | **Week 12** | 14.79  (3.95-44.10) | 9.43  (2.95-30.96) | | 11.70  (2.95-44.10) | | 0.449 |
|  |  | **Week 24** | 14.09  (6.27-43.79) | 13.72  (4.78-39.68) | | 13.91  (4.78-43.79) | | 0.349 |
|  |  | **Week 48** | 22.37  (2.50-48.85) | 26.46  (2.17-43.85) | | 24.27  (2.17-48.85) | | 0.533 |
| **CD8^+^CD38^+^ cells^b^**  percentage  median (range) | | **Baseline** | 29.38  (7.88-52.40) | 20.66  (2.99-64.70) | | 25.96  (2.99-64.70) | | 0.245 |
|  |  | **Week 4** | 20.34  (5.77-44.80) | 13.25  (4.08-35.30) | | 17.05  (4.08-44.80) | | 0.372 |
|  |  | **Week 12** | 15.69  (4.63-37.80) | 12.83  (1.93-33.20) | | 14.73  (1.93-37.80) | | 0.449 |
|  |  | **Week 24** | 13.13  (3.48-52.10) | 6.57  (2.32-26.30) | | 11.75  (2.32-52.10) | | 0.349 |
|  |  | **Week 48** | 9.78  (1.97-34.10) | 7.56  (1.42-33.00) | | 8.69  (1.42-34.10) | | 0.533 |
| **hs-CRP^c^**  mg/L  median (range) | | **Baseline** | 1.74  (0.22-42.31) | 0.93  (0.19-98.07) | | 1.46  (0.19-98.07) | | 0.337 |
|  |  | **Week 4** | 1.16  (0.23-46.53) | 2.00  (0.20-11.24) | | 1.28  (0.20-46.53) | | 0.337 |
|  |  | **Week 12** | 1.00  (0.09-5.88) | 2.05  (0.18-135.69) | | 1.08  (0.09-135.69) | | 0.295 |
|  |  | **Week 24** | 0.93  (0.17-49.31) | 1.92  (0.17-42.02) | | 1.12  (0.17-49.31) | | 0.273 |
|  |  | **Week 48** | 1.29  (0.12-6.92) | 0.94  (0.14-8.78) | | 1.19  (0.12-8.78) | | 1.000 |
| **D-dimer^d^**  µg FEU/mL  median (range) | | **Baseline** | 0.30  (0.20-1.50) | 0.30  (0.10-0.60) | | 0.30  (0.10-1.50) | | 0.863 |
|  |  | **Week 4** | 0.20  (0.10-0.80) | 0.30  (0.20-0.60) | | 0.30  (0.10-0.80) | | 0.612 |
|  |  | **Week 12** | 0.20  (0.10-0.70) | 0.30  (0.10-3.40) | | 0.20  (0.10-3.40) | | 0.220 |
|  |  | **Week 24** | 0.20  (0.10-0.50) | 0.20  (0.10-0.60) | | 0.20  (0.10-0.60) | | 0.798 |
|  |  | **Week 48** | 0.25  (0.10-0.60) | 0.30  (0.10-0.50) | | 0.30  (0.10-0.60) | | 0.331 |
| **Fibrinogen^e^**  mg/L  median (range) | | **Baseline** | 262  (125-358) | 240  (161-510) | | 262  (125-510) | | 0.419 |
|  |  | **Week 4** | 245  (58-374) | 214  (148-276) | | 235  (58-374) | | 0.109 |
|  |  | **Week 12** | 240  (114-338) | 206  (105-409) | | 232  (105-409) | | 0.325 |
|  |  | **Week 24** | 224  (123-441) | 240  (129-458) | | 225  (123-458) | | 0.918 |
|  |  | **Week 48** | 229  (87-460) | 241  (131-373) | | 236  (87-460) | | 0.843 |

Abbreviations: CVC, cenicriviroc; EFV, efavirenz; FEU, fibrinogen equivalent units.

^a^Calculated by Mann–Whitney U test.

^b^CD4^+^CD38^+^ and CD8^+^CD38^+^ cell percentages were available for 25 subject at week 24 in the CVC 200mg arm and 14 subjects at baseline and week 48 in the EFV 600mg arm.

^c^Week 12 hs-CRP was available for 25 subjects in the CVC 200mg arm.

^d^D-dimer was available for 24, 22, 25 and 22 subjects respectively at baseline, weeks 4, 24 and 48 (CVC 200mg arm); 12, 12, 9, 11 and 8 subjects respectively at baseline, weeks 4, 12, 24 and 48 (EFV 600mg arm).

^e^Fibrinogen was available for 25 subjects at week 24 in the CVC 200mg arm; 14, 14, 13, 14 and 14 subjects respectively at baseline, weeks 4, 12, 24 and 48 (EFV 600mg arm).
